# Selecting Dual Chamber or Single Chamber Implantable Defibrillators: What is the Golden Rule?

**Published:** 2003-10-01

**Authors:** Massimo Santini, Renato Ricci

**Affiliations:** Department of Cardiology, San Filippo Neri Hospital, Rome, Italy

**Keywords:** defibrillator, inappropriate shock, atrial fibrillation, heart failure, ventricular dysynchrony

## Introduction

During the last years, the implantation rate of dual chamber defibrillators (ICD) significantly increased worldwide. In 1999, the proportion of dual chamber ICD implants reached 30% in Europe and 50% in U.S.A. [1]. According to manufacturer data, in Italy, the ratio between implanted single chamber and dual chamber units decreased from 1.97 in 1999 to 1.86 in 2000 and 1.50 in 2001. Technological progress, demonstration of reliability and clinical efficacy of the new devices, combined with their smaller size, contributed to their wide acceptance. Nowadays, the matter to be debated is if all the patients in whom the atrium can be sensed and paced should receive a dual chamber ICD or if device selection should be individually evaluated according to different clinical profiles. As a matter of fact, criteria to identify the patients who may benefit more from dual chamber ICD have not been already defined. The theoretical advantages of dual chamber ICD include: improved discrimination between supraventricular and ventricular tachycardias, optimal treatment of symptomatic bradycardias (pre-existing, drug-induced or late developing), hemodynamic and antiarrhythmic benefits..

## Discrimination between supra-ventricular and ventricular arrhythmias

Superiority of dual chamber detection algorithms versus single chamber ICD in discriminating supraventricular from ventricular tachycardia has been a matter of debate since their introduction for clinical implantation. This issue is particularly challenging if we take into account that the addition of enhanced criteria in the third generation single chamber ICDs, such as tachycardia sudden onset and stability and ventricular electrogram width and morphology, significantly increased single chamber ICD specificity in tachycardia discrimination [2-5]. On the other hand, the weak point of such enhanced criteria is represented by decreased sensitivity in ventricular tachycardia detection [5,6]. Dual chamber ICDs have the capability of detecting atrial activity and matching atrial and ventricular patterns. Clinical studies using dual chamber ICDs showed specificity values as high as 80-90% combined with 100% sensitivity [7-12]. Nevertheless, inappropriate detection and therapy may still happen, mainly for "difficult arrhythmias". Hintringer et al [13] performed a comparison of the detection algorithms of four dual chamber ICD when dealing with a wide spectrum of tachyarrhythmias. In spite of some differences due the algorithms themselves, typical and atypical junctional tachycardia, orthodromic atrio-ventricular tachycardia and 1:1 atrial flutter represented the most challenging patterns. As a consequence, new more sophisticated algorithms are being developed to deal with this task. Appropriate sensing of atrial activity, either during sinus rhythm or during atrial tachyarrhythmias, and appropriate rejection of ventricular activity by the atrial lead is a critical issue to be dealt with. Deisenhofer et al [14] undertook a prospective, randomized study to compare the incidence of inappropriates therapies in patients treated with VVI-ICDs and DDD-ICDs. They enrolled 92 patients and concluded that DDD-ICD and VVI-ICD were equally safe and effective to treat life-threatening ventricular arrhythmias. Although DDD-ICDs theoretically allow better rhythm classification, the applied detection algorithms did not offer benefits in avoiding inappropriate therapies during supraventricular tachyarrhythmias. As a matter of fact, in the Deisenhofer series 75% of inappropriate therapies in the DDD-ICD group were due to atrial sensing problems, either oversensing or undersensing. This finding stresses the need of careful positioning of the atrial lead during implantation in order to combine optimal atrial electrogram amplitude with far field rejection. It has been suggested that positioning the atrial lead in the lateral atrial wall and selecting bipolar leads with short tip-to-ring distance may reduce far field incidence [15] [16]. In spite of that, atrial sensing problems may intermittently appear during the follow-up, also when they were not present at implant or during post-implant testing. A possible explanation for frequent intermittent atrial sensing problems may be the special filter settings in the atrial sensing channels of DDD-ICD, which differs substantially from those of DDD pacemakers. In fact, most detection algorithms in DDD-ICDs need correct and continuous atrial sensing with only short or even no blanking times. This may be difficult when taking into account low voltage atrial electrogram during atrial fibrillation and large ventricular far fields during paced ventricular beats.

## Hemodynamic issues

A emerging key point in debating optimal device selection is represented by the impact of single chamber and dual chamber ICD implantation on hemodynamics.

It has been demonstrated that, in patients with sinus bradycardia and/or atrio-ventricular conduction disturbances, physiologic pacing by sequential dual chamber stimulation and optimized, individually programmed, atrio-ventricular delay may offer major improvement in hemodynamics and clinical outcome, mainly when heart failure coexists [17-19]. On the other hand, during the last years it has been demonstrated that asynchronous ventricular activation induced by apical right ventricular pacing may induce major interventricular and/or intraventricular dysynchrony, which may deteriorate hemodynamics and impair myocardial metabolism [20,21]. Isovolumic contraction time and isovolumic relaxation time lenghtening may critically shorten diastolic filling, impairing cardiac output. Furthermore, delayed activation of the left ventricular lateral wall may lead to late contraction which happens after aortic valve closure, so that not only it does not contribute to stroke volume, but also impair diastolic filling [22].

Concern about the potential deleterious effect of unnecessary right ventricular pacing in ICD population is even greater than in pacemaker patients, when considering the higher prevalence of heart failure and left ventricular dysfunction in patients who need ICD implantation [23,24]. The recently published DAVID [25] (Dual Chamber and VVI Implantable Defibrillator) Trial dealt with this issue. Objective of the study was to determine the efficacy of dual chamber pacing compared with backup ventricular pacing in patients with standard indications for ICD implantation but without indications for antibradycardia pacing. The design of the study was a single-blind, parallel group, randomized, multicenter clinical trial. Five hundred and six patients candidates for ICD with left ventricular ejection fraction of 40% or less, no indications for antibradycardia pacing and no persistent atrial tachyarrhythmias, received a dual chamber ICD and were randomly assigned to have the ICD programmed to ventricular back-up pacing at 40/min or to dual chamber rate responsive pacing at 70/min. Main outcome measurement was the combined end point of time to death or first hospitalization for congestive heart failure. The study was early stopped by the Data and Safety Monitoring Board because the conditional power for the original alternative (DDDR-70 being better than VVI-40) was less than 10%. The VVI-40 group had fewer occurrences of the composite end point than DDD-70 group: one-year survival free was 83.9% versus 73.3% (relative hazard 1.61; 95% confidence interval 1.06-2.44, p< 0.03). Although the VVI-40 patients had fewer events, the component end points, either death or heart failure hospitalizations, did not individually reach statistical significance.

Some criticisms have been pointed out about the DAVID Trial. The study definitely demonstrated that right ventricular pacing is deleterious in patients with left ventricular dysfunction, but it cannot be concluded that single chamber ICD with back-up ventricular pacing is the most useful device for patients with heart failure. In spite of a large enrolled population, the follow-up was very short because of early stopping of the study. Only a minority of patients completed one year follow-up. Programming dual chamber rate responsive pacing at 70/min without long AV delay does not seem the best choice for patients without any indication for antibradycardia pacing. Unnecessary apical right ventricular pacing is probably the key to explain the higher event rates in DDD-70 arm. The Authors stressed that dual chamber pacing could be beneficial in heart failure patients since it may allow a wider use of drugs such as beta blockers which depress sinus and AV node function. As a matter of fact, there were no differences in drug regimen between the VVI and DDD arms after randomization as well as after 6-month follow-up. Finally, patients with atrial tachyarrhythmias were excluded from the study, so introducing a limitation in the clinical value of the study.

## Atrial fibrillation in ICD patients

Atrial fibrillation prevention and early treatment by dual chamber devices, mainly if equipped with atrial antitachycardia functions, may represent a major benefit in patients with heart failure. ICD patients actually show a high incidence of atrial tachyarrhythmias. It has been reported that 20% of them had atrial fibrillation before implantation and that during the life-span of the defibrillator more than 50% may develop atrial fibrillation [26]. Atrial fibrillation may lead to inappropriate ventricular shocks [27], ventricular arrhythmia induction [28], may impair hemodynamics and induce thromboembolic events or acute myocardial infarction, and has been identified as an individual predictor of poor prognosis [29,30]. Atrial antitachycardia functions available in some last generation dual chamber ICDs (pacing prevention algorithms and antitachycardia pacing) have been demonstrated to be effective in preventing and early treating atrial tachyarrhythmias. In our own experience [31], related to 112 patients receiving an ICD because of life threatening ventricular arrhythmias, followed on average for 1 year, anti-tachy-pacing efficacy was as high as 71% on atrial tachycardia and as 36% on atrial fibrillation. Shock success rate was 92% when delivered energy was adequately programmed, which means at least twice the atrial defibrillation threshold at implant. Similar results have been reported by others [32]. The impact of atrial prevention algorithms and atrial therapies on atrial fibrillation burden has been investigated by Friedman and coworkers [33]. They designed a study in which atrial fibrillation prevention and termination therapies were randomly programmed "on" or "off" for three months and then crossed over to the opposite arm for an additional 3 months. Fifty-two patients were studied. During the "on" period the arrhythmia burden (hours/month) significantly decreased: the mean burden from 58.5 to 7.8 and the median burden from 2.82 to 0.63. The mean burden reduction was 87%. The reduction in arrhythmia burden during the "on" period could be demonstrated also in the subgroup of patients (forty-one) in whom no shocks were delivered and only antitachy pacing therapies were applied.

Considering the major clinical impact of symptomatic atrial fibrillation in patients who are candidate for defibrillator implantation, a device equipped with atrial antitachycardia facilities may improve clinical outcome, by preventing acute heart failure, by decreasing inappropriate shocks, by reducing hospitalizations and by improving quality of life.

## Perspectives

The key point for single chamber or dual chamber ICD selection has been progressively switching from optimal tachycardia discrimination to the impact on hemodynamics and on atrial arrhythmia control. To this regard, few controlled data are available and perspectives randomized trials are strongly needed. The impact of device selection on the overall clinical outcome is the target of an ongoing trial [Dual Chamber & Atrial Tachyarrhythmias Adverse Events Study (DATAS), protocol in press [34]] aimed at comparing clinical benefits of dual chamber ICD with atrial antitachycardia functions with single chamber ICD. The primary end point will be the composite end-point resulting from all-causes mortality, invasive intervention, hospitalizations due to cardiovascular cause, inappropriate shocks and sustained symptomatic atrial tachyarrhythmias. The enrollment is going to be completed soon and the results will be available within the next two years.

On the other hand, new indications for ICD implantation in primary prevention of sudden death and introduction of triple chamber ICDs capable of delivering cardiac resynchronization therapy are going to change very soon the whole approach to ICD selection. The MADIT-2 trial [35] demonstrated that in patients with prior myocardial infarction and left ventricular ejection fraction < 30%, ICD implantion was able to reduce 2-year mortality by 31%. Rules for ICD selection in MADIT-2 patients are probably quite different from those applied for patients receiving an ICD in sudden death secondary prevention. A wider use of single chamber ICDs should be expected. First, accurate discrimination between supraventricular and ventricular tachycardia should be less meaningful in patients for whom therapy programming is focused mainly on treating fast ventricular tachycardia and ventricular fibrillation. Secondly, considering the large number of new potential candidates for ICD implantation, selecting a simpler and less costly device may improve the cost-effectiveness of ICD in primary prevention. Development of new low-cost single chamber devices, just capable of detecting ventricular fibrillation and delivering a limited number of shocks is expected for the next years. Such strategy will allow a wider protection of high risk population without an unacceptable increasing of the costs.

Cardiac resynchronization has been demonstrated to improve functional class, exercise tolerance and quality of life as well as to reduce hospitalisations due to worsening of heart failure [36,37] in patients with drug refractory heart failure with atrio-ventricular, inter-ventricular and intra-ventricular dysynchrony. Cardiac resynchronization may be combined with ICD. Considering the large number of ICD candidates with heart failure, drawing guidelines aimed to make the right choice for individual patients will be a major challenge for the next few years.
